# Progression of albuminuria and podocyte injury in focal segmental glomerulosclerosis inhibited by enhanced glycosphingolipid GM3 via valproic acid

**DOI:** 10.1038/s41598-023-49684-z

**Published:** 2023-12-15

**Authors:** Nagako Kawashima, Shokichi Naito, Masaki Nagane, Tadashi Yamashita, Ken-ichi Nakayama

**Affiliations:** 1 Department of Nephrology, School of Medicine, Kitasato University, 1-15-1 Kitasato, Minami, Sagamihara, Kanagawa 252-0374 Japan; 2 Laboratory of Biochemistry, School of Veterinary Medicine, Azabu University, 1-17-71 Fuchinobe, Chuo, Sagamihara, Kanagawa 252-5201 Japan; 3 National Institute of Advanced Industrial Science and Technology (AIST), 1-1-1 Umezono, Tsukuba, Ibaraki 305-8561 Japan

**Keywords:** Focal segmental glomerulosclerosis, Animal disease models

## Abstract

Focal segmental glomerulosclerosis, characterized by decreased numbers of podocytes in glomeruli, is a common cause of refractory nephrotic syndrome. Recently, we showed that enhanced glycosphingolipid GM3 expression after administration of valproic acid, an upregulator of *ST3GAL5/St3gal5*, was effective in preventing albuminuria and podocyte injury. We also revealed the molecular mechanism for this preventive effect, which involves GM3 directly binding nephrin that then act together in glycolipid-enriched membrane (GEM) fractions under normal conditions and in non-GEM fractions under nephrin injury conditions. Kidney disease is frequently referred to as a “silent killer” because it is often difficult to detect subjective symptoms. Thus, primary treatment for these diseases is initiated after the onset of disease progression. Consequently, the efficacy of enhanced levels of GM3 induced by valproic acid needs to be evaluated after the onset of the disease with severe albuminuria such as focal segmental glomerulosclerosis. Here, we report the therapeutic effect of enhanced GM3 expression induced via administration of valproic acid on albuminuria and podocyte injury after the onset focal segmental glomerulosclerosis in anti-nephrin antibody treated mice. Our findings suggest elevated levels of GM3 following treatment with valproic acid has therapeutic utility for kidney disease associated with severe albuminuria and podocyte injury.

## Introduction

The glomerular filtration barrier plays a critical role in normal kidney function and is formed by a slit diaphragm between the foot processes of neighboring podocytes. A decrease in the number of podocytes in glomeruli, which comprises the slit diaphragm, leads to focal segmental glomerulosclerosis (FSGS) (Fig. 5A). FSGS is a serious condition resulting in end stage renal disease (ESRD) that requires dialysis or a kidney transplant^1^. Thus, considerable research effort is directed towards studying the causes of FSGS as well as potential new treatments. Many studies in this field primarily investigated proteins implicated in FSGS^2^. More recently, the role of glycosphingolipids in kidney glomeruli has come under scrutiny. Specifically, the function of GM3, which is associated with vascular endothelial growth factor in normal kidney glomeruli, has been examined^3,4^. However, the role of GM3 in glomeruli, particularly podocytes, has yet to be fully established.

Recently, we analyzed nephrin, a key molecule in the slit diaphragm, and GM3, which localizes around the nephrin transmembrane domain of podocytes. In these experiments we used a podocytopathy mouse model, which is induced by administration of anti-nephrin antibody (anti-Nphs Ab). Notably, we found that pretreatment of valproic acid (VPA), which activates the GM3 synthase gene (*ST3GAL5/St3gal5*), dramatically inhibited albuminuria and podocyte injury in the podocytopathy mouse model^5^. Thus, the onset of albuminuria and podocyte injury can be prevented by enhanced glycosphingolipid GM3 expression. Furthermore, to clarify the mechanism by which this preventive effect occurs, we experimented with several cell lines. First, we performed immunoprecipitation experiments to examine whether escape from nephrin injury by enhanced GM3 expression was due to interaction between GM3 and nephrin, a component of the slit diaphragm. These experiments revealed a direct interaction between GM3 and nephrin. Furthermore, we investigated whether GM3 acts together with nephrin in raft (GEM) fractions or non-raft (non-GEM) fractions. The results showed that under normal conditions both nephrin and GM3 localized in raft (GEM) fractions, but under abnormal membrane conditions, such as when anti-Nphs Ab binds to nephrin, raft (GEM) fractions become disordered and nephrin/GM3 shift to non-raft (non-GEM) fractions. Thus, these experiments also indicated that nephrin could escape injury induced by various extracellular stimuli via enhanced levels of GM3. Additionally, when the same experiment was conducted using *St3gal5* knockout (*St3gal5*^*−/−*^) mice, not only did anti-Nphs Ab administration induce irreversible albuminuria, but podocyte injury was not inhibited by treatment with VPA^5^.

Based on these findings, we concluded that this preventive mechanism is due to the enhancement of GM3 expression via activation of *ST3GAL5/St3gal5* by VPA, which stabilizes nephrin expression and maintains healthy podocytes. We have also found that albuminuria correlated inversely with GM3 expression in patients with podocyte injury due to slit diaphragm damage (minimal change disease: MCD, FSGS)^6^.

To develop an effective treatment for FSGS, the efficacy of GM3 after the onset of pathological conditions must be established. However, it is unclear whether the enhancement of GM3 expression by VPA has a therapeutic effect following the onset of albuminuria and podocyte injury. Therefore, in this study, we investigated the upregulation of GM3 expression by administration of VPA after the onset of FSGS using model mice and examined the therapeutic outcome by assessing albuminuria, glomerulosclerosis, and loss of podocytes. Our results indicate that VPA-mediated upregulation of *St3gal5* can inhibit the progression of kidney disease with severe albuminuria, such as FSGS. Thus, we propose that maintaining sufficient levels of GM3 expression in podocytes is effective not only for the prevention but also the treatment of podocyte injury.

## Results

### Inhibition of FSGS progression by GM3 via valproic acid administration

**Figure 1:**
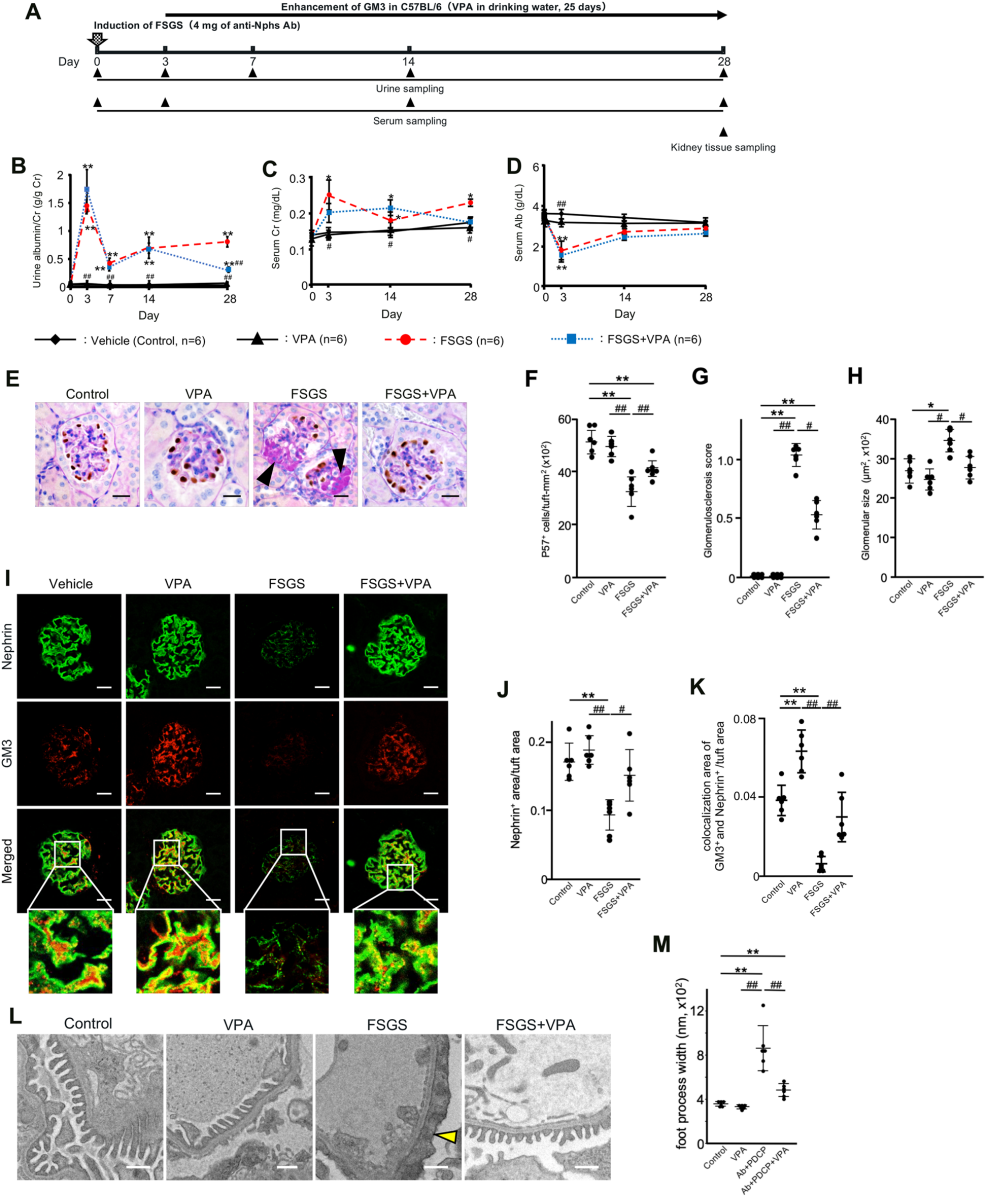
Therapeutic effect by GM3 via administration of VPA in anti-nephrin antibody-induced FSGS mice. (**A**) Schedule for FSGS therapeutic test using valproic acid (VPA). (**B**) Line graph showing urine albumin to creatinine, (**C**) serum creatinine, (**D**) serum albumin. Vehicle (Control), VPA administered mice (VPA), 4 mg of anti-nephrin antibody (anti-Nphs Ab)-induced FSGS (FSGS), and 4 mg of anti-Nphs Ab-induced FSGS + VPA administered (FSGS + VPA) mice are shown as a solid line with diamond, solid line with triangle, dashed red line with red circle and dotted blue line with blue square, respectively. *P < 0.05; **P < 0.01 vs. control, ^#^*P* < 0.05; ^##^*P* < 0.01 vs. FSGS. (**E**) p57 (podocyte marker, brown) and PAS staining of glomeruli. Arrowheads showing glomerulosclerosis. Scale bars: 20 µm. (**F**) Scatter diagram showing p57^+^ cells/tuft, (**G**) glomerulosclerosis score, and (**H**) glomerular size, respectively. Twenty glomeruli per mouse were analyzed in each dot. *P < 0.05; **P < 0.01 vs. control, ^#^*P* < 0.05; ^##^*P* < 0.01 vs. FSGS. (**I**) Immunofluorescence staining of glomeruli. Scale bars: 20 µm. Nephrin (green) and GM3 (red) merged areas (yellow) highlighted in enlarged images. (**J**) Scatter diagram showing nephrin fluorescence area/tuft area, (**K**) GM3 and nephrin merged fluorescence area/tuft area, respectively. **P < 0.01 vs. control, ^#^*P* < 0.05; ^##^*P* < 0.01 vs. FSGS. (**L**) Images of podocyte foot processes obtained by transmission electron microscopy. Yellow arrowhead showing area of foot process effacement. Scale bars: 10 µm. (**M**) Scatter diagram showing quantification of foot process effacement. **P < 0.01 vs. control, ^##^*P* < 0.01 vs. FSGS. Statistical analysis was performed for each group of mice (n = 6).

Therapeutic tests were performed using FSGS model mice induced by administration of 4 mg of anti-Nphs Ab (Fig. 1A). In the late phase of induction of FSGS progression (day 7 onwards), brought about the loss of podocytes^7^, albuminuria gradually increased in FSGS mice (Fig. 1B). However, albuminuria decreased again after day 14 in FSGS + VPA mice compared with FSGS mice (Fig. 1B). Although FSGS and FSGS + VPA mice had higher levels of serum creatinine compared with the control, FSGS + VPA mice displayed a gradual improvement by day 28 (Fig. 1C). Specifically, serum albumin levels for FSGS + VPA were almost the same as FSGS on day 3, but then recovered to levels similar to control mice (Fig. 1D). VPA administration alone had no effect on mice (Fig. 1B–D). Moreover, the number of p57-positive cells/tufts decreased in FSGS mice, compared with control, VPA and FSGS + VPA mice (Fig. 1E–F). In spite of FSGS induction by anti-Nphs Ab, FSGS + VPA mice showed reduced levels of glomerulosclerosis and smaller glomerular size compared to FSGS mice (Fig. 1G–H). Although the levels of both nephrin and GM3 in FSGS mice were drastically reduced, the expression of both molecules in FSGS + VPA mice remained at almost the same levels as the control mice (Fig. 1I–K). Foot process effacement and podocyte depletion had clearly occurred in FSGS mice (Fig. 1L–M). In FSGS + VPA mice, however, foot processes were maintained (Fig. 1L–M). Taken together, our results showed (i) nephrin and GM3 levels were clearly maintained in FSGS + VPA mice by comparison with FSGS mice, and (ii) elevated levels of GM3 via administration of VPA inhibited the progression of FSGS induced by anti-Nphs Ab (Fig. 1).

### Influence of anti-Nphs Ab and VPA on morphology in anti-Nphs Ab induced-FSGS mice kidney

**Figure 2:**
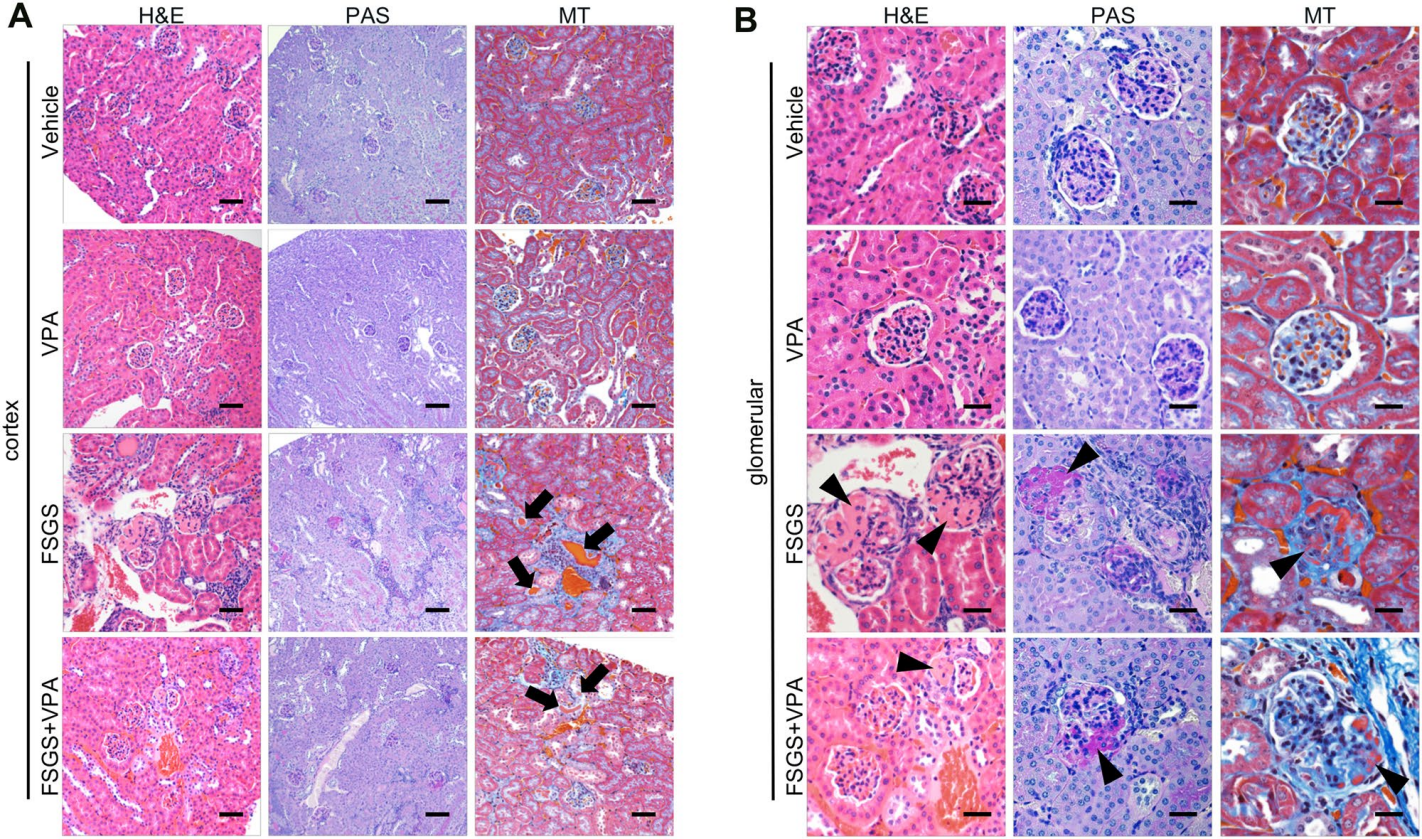
Influence of anti-Nphs Ab and VPA on morphology in anti-Nphs Ab-induced FSGS mice kidney glomeruli. (**A**) Histological analysis after H&E (HE), PAS and Masson’s trichrome (MT) staining of mouse kidney cortex and (**B**) glomeruli. Control (Vehicle), VPA administered mice (VPA), 4 mg of anti-Nphs Ab-induced FSGS (FSGS), and FSGS + VPA administered (FSGS + VPA) mice. Arrows highlight urinary casts within expanded tubules. Arrowheads indicate glomerulosclerosis. Scale bars: 100 µm (**A**), 20 µm (**B**).

To analyze morphological changes in mice kidney, Masson’s trichrome (MT) staining in parallel with hematoxylin and eosin (H&E) and periodic acid-Schiff (PAS) staining were performed. Glomerulosclerosis and intratubular casts, which are also observed in the mouse model of Adriamycin nephrosis^8^, were clearly observed in FSGS mice by comparison with control or VPA mice (arrows in Fig. 2A, arrowheads in Fig. 2B). The same morphological changes that occurred in FSGS mice were also observed in FSGS + VPA mice (Fig. 2A,B). However, in FSGS + VPA mice, the changes were slight by comparison to FSGS mice.

### Integrin β1 and GM3 expression in anti-Nphs Ab-induced FSGS mice

**Figure 3:**
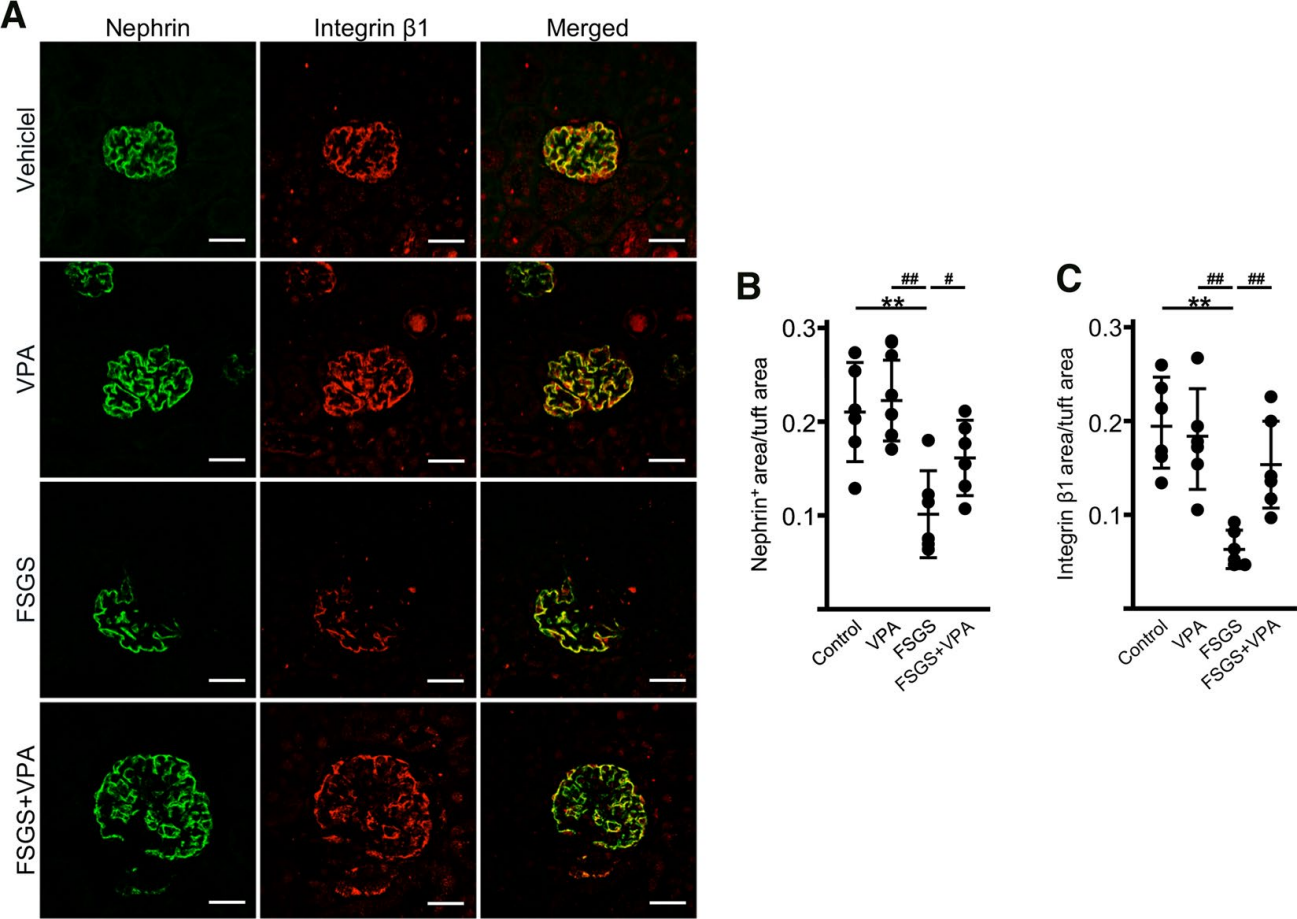
Nephrin and integrin β1 expression in anti-Nphs Ab-induced FSGS mice kidney glomeruli. (**A**) Immunofluorescence staining images of nephrin (green) and integrin β1 (red) in treated mice. Control (Vehicle), VPA administered mice (VPA), 4 mg of anti-Nphs Ab treated (FSGS), and FSGS + VPA administered (FSGS + VPA) mice. Scale bars: 20 µm. (**B**) Scatter diagram showing nephrin fluorescence area/tuft area, (**C**) Integrin β1 fluorescence area/tuft area, respectively. Twenty glomeruli per mouse were analyzed in each dot. **p < 0.01 vs. control. ^##^*p* < 0.01, ^#^*p* < 0.05 vs. FSGS, respectively. Statistical analyses was performed for each group of mice (n = 6). Immunofluorescence staining was performed for nephrin or GM3 and data was analyzed using scatter diagrams for the same mice used in (**A**) and Fig. 1I–K.

Based on localization analysis of cell membrane molecules^5^, we reasoned that anti-Nphs Ab and VPA treatment might affect the expression of integrin β1, which is involved in cell adhesion. Therefore, to test this hypothesis, expression of integrin β1, GM3 and nephrin in the glomerulus of various treated mice were analyzed by immunofluorescence staining (Fig. 3). Due to the characteristics of the antibodies, the co-staining of integrin β1 and nephrin was performed using paraffin sections (Fig. 3A), and that of GM3 and nephrin was performed using frozen tissues (as shown in Fig. 1I). Our results showed that nephrin, GM3 and integrin β1 were readily detected in untreated mice, but the levels of all these molecules were markedly reduced in anti-Nphs Ab-induced FSGS mice (Fig. 1I–K, Fig. 3A–C). However, FSGS + VPA mice displayed levels of integrin β1 and GM3 similar to those observed in control mice (Figs. 1I–K, 3A–C). Thus, the therapeutic effect of enhanced levels of GM3 brought about by treatment with VPA in FSGS mice results from (i) maintenance of nephrin expression, and (ii) maintenance of integrin β1, which may be involved in podocyte adhesion (Fig. 5B). These results are consistent with the proposal that VPA blocks podocyte depletion from the glomeruli.

### No therapeutic effect with VPA in podocytopathy induced GM3 synthase gene knockout (*St3gal5*^−/−^) mice

**Figure 4:**
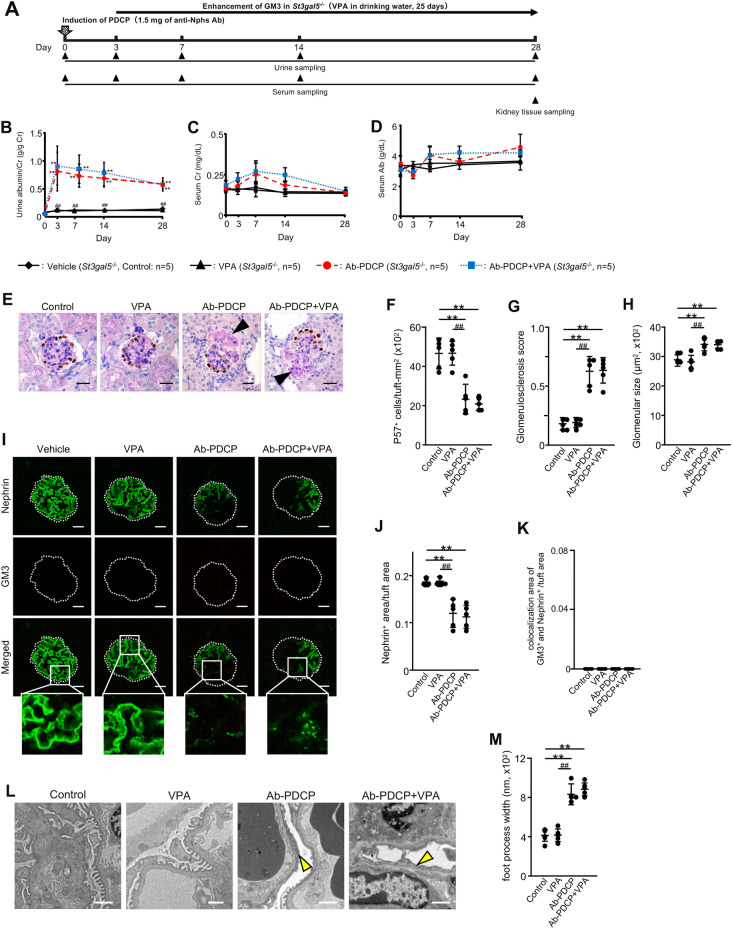
No therapeutic effect by GM3 via administration of VPA in young anti-nephrin antibody-induced podocytopathy GM3 synthase gene knockout (*St3gal5*^−/−^) mice. (**A**) Schedule for podocytopathy (PDCP) therapeutic test using VPA. (**B**) Line graph showing urine albumin to creatinine, (**C**) serum creatinine, (**D**) serum albumin. Vehicle (Control), VPA administered mice (VPA), 1.5 mg of anti-Nphs Ab-induced podocytopathy (Ab-PDCP), and 1.5 mg of anti-Nphs Ab-induced PDCP + VPA administered (Ab-PDCP + VPA) mice are shown as a solid line with diamond, solid line with triangle, dashed red line with red circle and dotted blue line with blue square, respectively. **P < 0.01 vs. control, ^##^*P* < 0.01 vs. Ab-PDCP. (**E**) p57 (podocyte marker, brown) and PAS staining of glomeruli. Arrowheads showing glomerulosclerosis. Scale bars: 20 µm. (**F**) Scatter diagram showing p57^+^ cells/tuft, (**G**) glomerulosclerosis score, and (**H**) glomerular size, respectively. Twenty glomeruli per mouse were analyzed in each dot. **P < 0.01 vs. control, ^##^*P* < 0.01 vs. Ab-PDCP. (**I**) Immunofluorescence staining of glomeruli. Scale bars: 20 µm. Nephrin (green) and GM3 (red) were not merged in enlarged images. (**J**) Scatter diagram showing nephrin fluorescence area/tuft area, (**K**) GM3 and nephrin merged fluorescence area/tuft area, respectively. **P < 0.01 vs. control, ^##^*P* < 0.01 vs. Ab-PDCP. (**L**) Images of podocyte foot processes obtained by transmission electron microscopy. Yellow arrowhead showing area of foot process effacement. Scale bars: 10 µm. (**M**) Scatter diagram showing quantification of foot process effacement. **P < 0.01 vs. control, ^##^*P* < 0.01 vs. Ab-PDCP. Statistical analysis was performed for each group of mice (n = 6).

Therapeutic tests were performed using a podocytopathy (PDCP) model induced by administration of 1.5 mg of anti-Nphs Ab in *St3gal5*^−/−^ mice (Fig. 4A). In contrast to control and VPA treated *St3gal5*^−/−^ mice, both Ab-PDCP and Ab-PDCP + VPA treated *St3gal5*^−/−^ mice displayed elevated levels of albuminuria by day 3 after administration of anti-Nphs Ab, which continued until day 28 (Fig. 1B). Moreover, the level of albuminuria in Ab-PDCP + VPA treated *St3gal5*^−/−^ mice was the same as in Ab-PDCP treated *St3gal5*^−/−^ mice (Fig. 4B). Albuminuria levels were almost normal in the control and VPA treated groups (Fig. 4B). There were no significant differences in serum creatinine and serum albumin among the four groups (Fig. 4C,D). As for the number of podocytes, glomerulosclerotic score, and glomerular size, there was an increase in sclerotic lesions, a decrease in podocyte count, and glomerular hypertrophy in Ab-PDCP treated *St3gal5*^−/−^ mice compared to the control and VPA treated mice (Fig. 4E–H). These glomerular morphological changes caused by PDCP via anti-Nphs Ab were the same in Ab-PDCP + VPA treated *St3gal5*^−/−^ mice (Fig. 4E–H). Nephrin was clearly expressed in control and VPA treated mice (Fig. 4I–K). However, both Ab-PDCP and Ab-PDCP + VPA treated *St3gal5*^−/−^ mice showed decreased expression of nephrin (Fig. 4I–K). GM3 was not expressed in all groups due to the lack of the *St3gal5* gene (Fig. 4I, K). The same degree of podocyte foot process effacement was observed in both Ab-PDCP and Ab-PDCP + VPA treated mice along with many microvilli within the Bowman's space (Fig. 4L,M). However, these observations were not found in control and VPA treated mice (Fig. 4L,M).Unlike the results of wild-type mice (Fig. 1), VPA administration had no therapeutic effect on proteinuria and glomerular injury in *St3gal5*^−/−^ mice (Fig. 4). The results obtained in this study are also consistent with our previous finding^5^ that VPA has no preventive effect on PDCP in *St3gal5*^−/−^ mice. Taken together, the results of the VPA therapeutic efficacy study also suggest that GM3 expression is essential for normal podocyte function.

## Discussion

We previously reported that pretreatment with VPA can block the onset of proteinuric kidney disease caused by damage to the slit diaphragm, and that this preventive effect is due to enhanced GM3 expression in podocytes via upregulation of *ST3GAL5/St3gal5*^5^. However, kidney disease is often referred to as a "silent killer" because subjective symptoms are difficult to detect, and primary treatment for most conditions is only initiated after kidney disease onset^9^. In general, most low molecular weight drugs can be administered orally because they are resistant to degradation by digestive enzymes in the gastrointestinal tract and have good cell membrane permeability^10,11^. Therefore, the efficacy of VPA via oral administration using FSGS and podocytopathy induced model mice after the onset of the kidney disease also needs to be evaluated.

**Figure 5:**
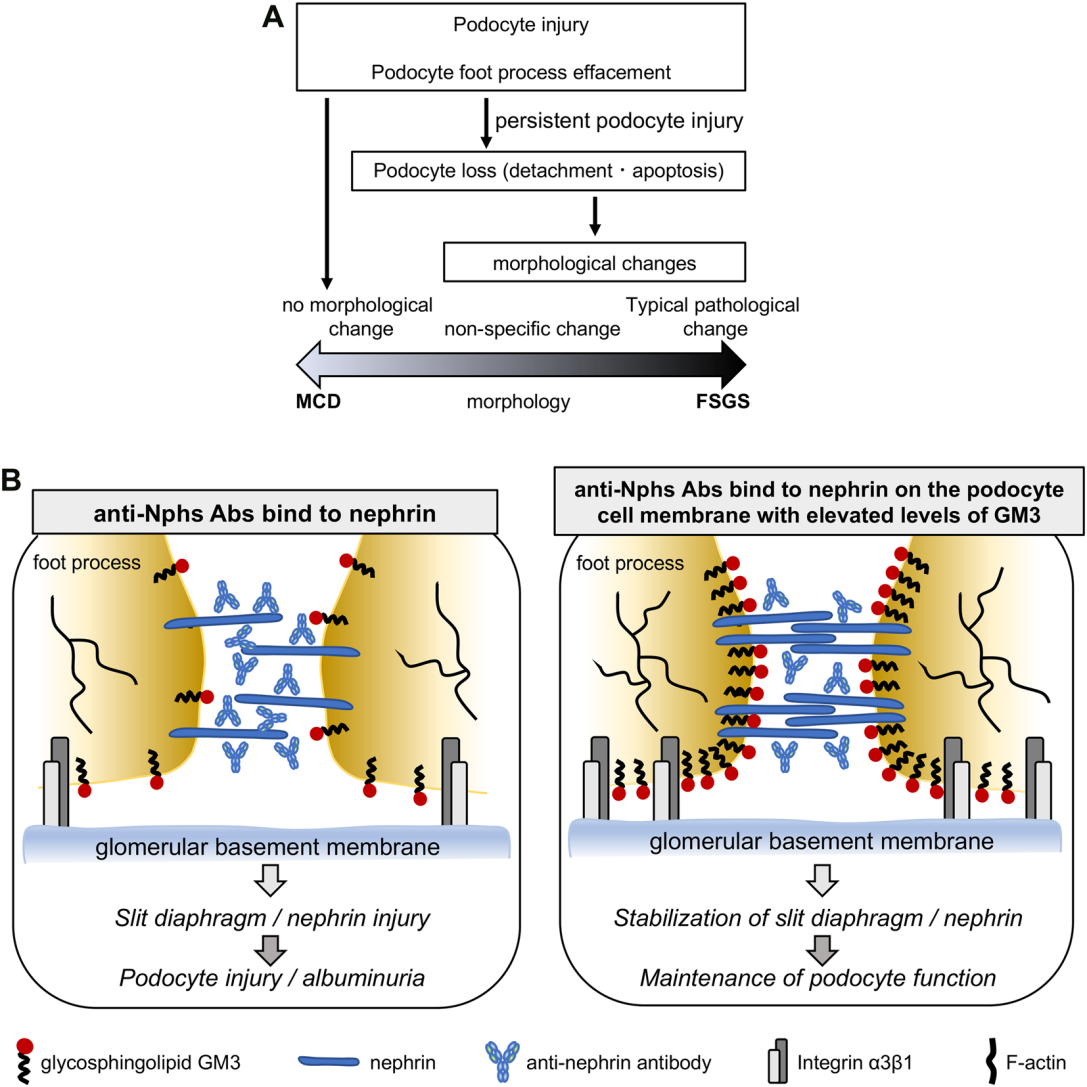
Mechanism of podocyte injury and model depicting how enhanced levels of GM3 induced by administration of VPA inhibits albuminuria and podocyte injury. (**A**) Flowchart depicting the relationship between MCD and FSGS. (**B**) VPA upregulates the expression of *St3gal5*, which encodes GM3 synthase. Enhanced GM3 synthase gene expression leads to an increase in the level of GM3 at the cell surface. Consequent changes to the underlying actin cytoskeleton help maintain a healthy morphology, reducing the severity of albuminuria and podocyte injury.

In this study, we induced FSGS and podocytopathy model mice by injection of anti-Nphs Ab, which causes albuminuria^5,7^, followed by administration of VPA in drinking water to analyze the therapeutic effects of this compound. There was no difference in albuminuria level between the FSGS and FSGS + VPA treated in wild-type mice for the first 7 days after injection of anti-Nphs Ab (4 mg) (Fig. 1) due to severe injury to the podocytes. In addition, when the serum albumin concentration data were taken into consideration, the similarity of albuminuria and serum albumin concentrations in the FSGS and FSGS + VPA mice on day 3 suggested that the injury level to the cell membrane was almost the same at the start point of VPA administration (Fig. 1B,D). Administration of anti-Nphs Ab leads to progression of FSGS caused by the loss of podocytes^7^. However, from day 14 onwards FSGS progression diminished for the in FSGS + VPA mice as observed by the gradual decrease in albuminuria (Fig. 1B). This result indicates that VPA treatment after the onset of FSGS can suppress the degree of albuminuria in the longer term. Moreover, therapeutic administration of VPA not only inhibits the progression of glomerulosclerosis but also impedes the development of tubulointerstitial lesions (Fig. 2). Integrin β1, which is known to be highly expressed in the basement membrane of glomeruli^12^, was found to remain following VPA administration (Fig. 3A,C), albeit at a markedly reduced level as a result of podocyte injury induced by anti-Nphs Ab (Fig. 3A,B). Thus, VPA-mediated enhanced GM3 expression robustly maintains the cell membrane of podocytes despite the injury mediated by anti-Nphs Ab. As a consequence of VPA administration, residual levels of actin polymerization^5^ and integrinβ1 expression levels appear to be important in preventing podocyte detachment and the transition to severe glomerular damage (Fig. 5B). This suggests that GM3 may be involved not only in interaction with nephrin but also indirectly with integrinβ1, which prevents podocyte detachment and shedding.

To confirm that these therapeutic effects were due to VPA-mediated enhancement of GM3 expression, we also attempted to conduct the same schedule test as described in Fig. 1A using GM3 synthase gene knockout (*St3gal5*^−*/*−^) mice. Specifically, the schedule involved injection of 4 mg of anti-Nphs Ab to bring about the onset of FSGS followed by administration of VPA in drinking water 3 days later. However, the therapeutic effect of VPA could not be assessed because *St3gal5*^*-/-*^ mice died on day 1 after injection of a high dose (4 mg) of anti-Nphs Ab. Therefore, we performed the therapeutic study of VPA in FSGS induced wild-type mice using a high dose (4 mg) of anti-Nphs Ab (Fig. 1), but also in PDCP induced *St3gal5*^−/−^ mice using a low dose (1.5 mg) of the same antibody (Fig. 4A). However, 16.7% of *St3gal5*^−/−^ mice died within 3 days of anti-Nphs Ab administration, even though the amount of antibody was reduced to a low dose (1.5 mg) from a high dose (4 mg). We concluded that the cause of death may have been hypovolemic shock associated with nephrotic syndrome. The degree of glomerular lesions, podocyte injury, and nephrin expression in the surviving mice were all similar among the Ab-PDCP treated and Ab-PDCP + VPA treated groups (Fig. 4B–M). The results of this VPA therapeutic study using GM3 synthase gene knockout (*St3gal5*^−*/*−^) mice is also completely consistent with the results of the VPA prevention study^5^, which showed that VPA had no effect in *St3gal5*^−/−^ mice (Fig. 4).

We previously reported that young *St3gal5*^−/−^ mice (13 week-old) showed only very minor changes in the glomeruli, and obvious symptoms such as the loss of podocytes were not reflected in kidney glomeruli^5^. *St3gal5*^*−/−*^ mice had a normal appearance with no apparent effect on survival in the pathogen-free environment. Nonetheless, once positively stimulated by anti-Nphs Abs the transition to an irreversible and severe disorder in these mice was inevitable^5^. Thus, GM3 synthase gene knockout (*St3gal5*^*−/−*^) mice are much more responsive to external stimuli than wild-type mice^5^. Indeed, a previous report^13^ stated that “because the expression of glycosphingolipids is controlled by more than one mechanism, the concentration of glycosphingolipids displayed on the surface of the cell may be much more sensitive to subtle shifts in the environment than, say, the concentration of a protein”. Administration of a high dose of anti-Nphs antibody (i.e., 4 mg) is a large external stimulus. Although wild-type mice did not die after being given a high dose of anti-Nphs antibody^7^, *St3gal5*^*−*/*−*^ mice were more susceptible to hypovolemic shock associated with nephrotic syndrome. Furthermore, there was no transient decrease of albuminuria in *St3gal5*^*−*/*−*^ mice after injection of 1.5 mg of anti-Nphs antibody, in contrast to wild-type mice in both this study (Fig. 4) and a previous study^5^. Taken together, these observations suggest that GM3 expression is important for the prevention and treatment of albuminuria and glomerular injury i.e., essential for the maintenance of normal kidney function.

This study has found that glycosphingolipid GM3 can be used not only to prevent albuminuria and glomerulosclerosis, as previously reported^5,6^, but also to treat these conditions after disease onset. Of particular importance is the therapeutic effect observed when GM3 levels were restored in FSGS mice with severe albuminuria (Fig. 1). Recently, we reported the therapeutic effects of VPA in diabetic nephropathy model mice, and that VPA and proteinuria were inversely correlated in patients with diabetic nephropathy^14^. It will be interesting to investigate the potential prevention effect and treatment efficacy of GM3 via VPA administration for other proteinuric kidney diseases^1^. Such studies will provide important information for future drug discovery aimed at treating or preventing proteinuric kidney diseases, which are currently becoming increasingly more common across the world.

## Methods

### Reagents and antibodies

Reagent and antibodies are listed in Supplementary Table S1. The protocol used for the production of anti-mouse nephrin (C-terminus) antibody (anti-cNphs Ab) was described previously^5^. In brief, anti-cNphs Ab was raised against the C-terminus of nephrin in rabbits using synthetic peptides conjugated to keyhole limpet hemocyanin. The sequence of the peptide antigen corresponded to the C-terminal region of mouse nephrin; GEPGSLPFELRGHLVC(C). Antibodies from antisera were purified using peptide antigen conjugated to Sepharose 6B. The anti-cNphs Ab was further purified by passage through nephrin C-terminus peptide conjugated to Sepharose 6B for removal of cross-reactive antibodies.

### Animal studies

C57BL/6N mice (male, 6 week-old, 17–19 g) were purchased from CLEA Japan Inc. (Tokyo, Japan) and GM3 synthase gene knockout (*St3gal5*^−/−^) mice (male, 13 week-old, 18–20 g)^5,15^ were housed in metabolic cages under specific-pathogen-free conditions and fed a standard chow. Mice were placed under 2–5% isoflurane anesthesia for euthanasia.

### Generation of FSGS and podocytopathy mice

For in vivo testing, mice were randomized into four groups (n = 5 or 6 for each group) as follows: (i) control group, (ii) VPA treated group, (iii) anti-Nphs Ab-induced FSGS or podocytopathy group, (iv) VPA treated anti-Nphs Ab-induced FSGS or podocytopathy group. We used an anti-mouse nephrin polyclonal antibody (anti-Nphs Ab) raised in rabbits as previously reported^5,7^. To induce FSGS or podocytopathy, mice were injected with a single aliquot of 4 or 1.5 mg of anti-Nphs Ab via the tail vein^7^, respectively. VPA was administered as 4 mM VPA in drinking water (100 mg/kg/day human equivalent dose, which is known to be safe in human)^16^. Kidney tissues from the four groups of mice were sampled on day 28 after anti-Nphs Ab administration and used for various analyses.

### Biochemical studies

For FSGS therapeutic tests, urine and blood were sampled on each of the following days: 0, 3, 7, 14, 28 and 0, 3, 14, 28 after anti-Nphs Ab administration, respectively. Each serum was prepared from blood by centrifugation at 1200×*g* for 20 min at 4 °C. Urine albumin, urine creatinine, serum creatinine, and serum albumin were measured using Albuwell M (#1011, Ethos Biosciences, Logan Township, NJ, USA), Creatinine Companion kit (#1012, Ethos Biosciences), Creatinine Assay kit Quantichrom (#DICT-500, BioAssay Systems, Hayward, CA, USA), and A/G B-test wako (#274-24,301, FUJIFILM Wako, Osaka, Japan), respectively.

### Tissue staining

In brief, kidney tissues were subject to p57 and periodic acid-Schiff (PAS) staining, H&E staining, Masson’s trichrome (MT) staining, and glomerulosclerosis were assessed as described previously^5,7,14,17,18^. Immunofluorescence staining of nephrin and GM3 was performed as previously outlined^5^. Pathological images and fluorescence images were obtained by optical microscopy (BX51; Olympus, Tokyo, Japan) and confocal laser-scanning microscopy (LSM710; Carl Zeiss, Oberkochen, Germany), respectively. The images were analyzed using ImageJ software (http://imagej.nih.gov/ij/) and ZEN imaging software (Carl Zeiss).

### Electron microscopy

Mouse kidney cortex samples were fixed in 0.1 M sodium cacodylate buffer (pH 7.4) containing 2.5% glutaraldehyde for 3 h at 4 °C. After washing in 0.1 M sodium cacodylate buffer (pH 7.4) the samples were fixed in 0.1 M sodium cacodylate buffer (pH 7.4) containing 2% OsO_4_ for 1 h. Following dehydration with ethanol, the buffer was replaced with *n*-butyl glycidyl ether (QY-1; Nisshin EM, Tokyo, Japan) and the samples embedded in epoxy resin (Quetol 812; Nisshin EM). Ultrathin Sects. (80 nm-thick) of renal cortex were prepared and stained with 3% uranyl acetate and lead citrate. Samples were analyzed using a transmission electron microscope (H-7650; Hitachi, Tokyo, Japan) fitted with a CCD camera (#Veleta; Olympus, Tokyo, Japan).

### Statistical analysis

Data were analyzed as the mean ± SEM. Statistical comparisons between two groups were evaluated by Mann–Whitney’s U-test. Multiple-group comparisons were evaluated using 1-way ANOVA followed by Tukey’s test. Statistical analysis was performed by StatFlex Ver.7 (Artech, Osaka, Japan). A *P*-value of less than 0.05 was considered statistically significant.

### Study approval

All animal experiments were approved by the Ethical Review Committee for Animal Experiments of Kitasato University School of Medicine (#2018-006, #2019-072, #2020-016) and Azabu University of Veterinary Medicine (#201204-2). DNA experiments were approved by the Ethical Review Committee for Recombinant DNA Experiments of Kitasato University School of Medicine (#3837). All experiments were performed in accordance with relevant guidelines and regulations and the study is reported in accordance with ARRIVE guidelines.

### Supplementary Information

Supplementary Information.

## Data Availability

The data that support the findings of this study are available in the main figures and the Supplementary Material of this article and are also available upon reasonable request.
