# Supplementary material for: Progression of albuminuria and podocyte injury in focal segmental glomerulosclerosis inhibited by enhanced glycosphingolipid GM3 via valproic acid
Source: Sci Rep. 2023 Dec 15;13:22487. doi: 10.1038/s41598-023-49684-z (PMC10728181; doi:10.1038/s41598-023-49684-z)
Supplement: Supplementary file 1 — Supplementary Information. [file 41598_2023_49684_MOESM1_ESM.pdf]

Supplementary Information

Progression of albuminuria and podocyte injury in focal segmental glomerulosclerosis inhibited by enhanced glycosphingolipid GM3 via valproic acid

Nagako Kawashima, Shokichi Naito, Masaki Nagane, Tadashi Yamashita and Ken-ichi Nakayama

This file included:

Supplementary Table S1

Supplementary Result

Supplementary Figure S1-2 and Figure Legend S1-2

Supplementary References

Supplementary Table

Supplementary Table S1 Reagent, Antibodies for immunofluorescence

**Name**	**Source**	**Type**
Valproic acid sodium salt	FUJIFILM Wako, #193-18352	powder
Anti-nephrin antibody	Original [1] (used for induction of FSGS *in vivo* tests)	Rabbit, polyclonal
Anti-nephrin (C-terminus) antibody (anti-cNphs Ab)	Original [2] (established in this study, used for detection of nephrin *in vivo* tests)	Rabbit, polyclonal
Anti-p57 antibody (H-91)	Santa Cruz Biotech. #sc-8298	Rabbit, polyclonal
Anti-GM3 antibody (GMR6)	Tokyo Chemical Industry, #A2582	Mouse, monoclonal
Anti-GM2 antibody (MK1-16)	Tokyo Chemical Industry, #A2575	Mouse, monoclonal
Anti-GD3 antibody (GMR19)	Tokyo Chemical Industry, #A2579	Mouse, monoclonal
Alexa Fluor555-Phalloidin	Thermo Fisher Scientific, #A34055	-

Alexa488-Rabbit anti-Mouse IgG	Thermo Fisher Scientific, #A27023	Rabbit, polyclonal
Alexa568-Goat anti-Mouse IgG	Thermo Fisher Scientific, #A11004	Rabbit, polyclonal
Alexa Fluor 488-Goat anti-Mouse IgM	Life Technologies, #A-21042	Goat, polyclonal
Alexa Fluor 568-Goat anti-Mouse IgM	Life Technologies, #A-21043	Goat, polyclonal

Supplementary Result

GM3 specific biosynthesis via VPA in the ganglioside biosynthetic pathway

To investigate VPA specificity for enhancement of GM3 expression in glycosphingolipid biosynthesis via induction of ST3GAL5, the levels of related gangliosides GM2 (GalNAcβ4[NeuAcα3]Galβ4GlcβCer) and GD3 (NeuAcα2-8NeuAcα2-3Galβ4Glc1Cer) were examined by immunofluorescence staining (Supplemental Figure S1A, B). Both GM2 and GD3 expression was very low, and no significant differences were observed in the four groups of mice (Control, VPA, FSGS, FSGS+VPA). These results suggest that VPA and anti-Nphs Ab exert a specific effect on GM3 biosynthesis.

Supplemental Figure S1

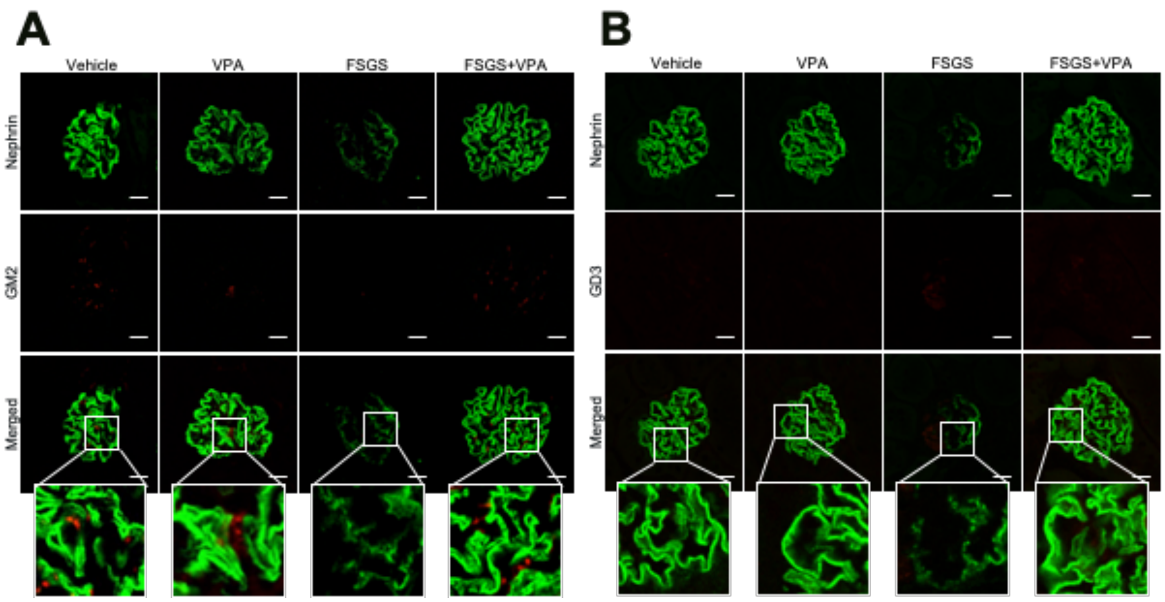

Supplemental Figure S1. Influence of anti-Nphs Ab and VPA on ganglioside biosynthesis in FSGS mice

A: Immunofluorescence staining images of nephrin and GM2 in various treated mice. **B:** Immunofluorescence staining images of nephrin and GD3 in the same tissues as in **A**. Scale bars: 20 µm. Vehicle (Control), VPA administrated mice (VPA), anti-nephrin Ab (anti-Nphs Ab) treated (FSGS), and anti-Nphs Ab+valproic acid (VPA) treated (FSGS+VPA) mice. Nephrin (green) and GM3 (red) highlighted in enlarged images.

Supplementary References

1 Takeuchi, K. *et al.* New Anti-Nephrin Antibody Mediated Podocyte Injury Model Using a C57BL/6 Mouse Strain. *Nephron* 138, 71-87, doi:10.1159/000479935 (2018).

2 Kawashima, N. *et al.* Glycosphingolipid GM3 prevents albuminuria and podocytopathy induced by anti-nephrin antibody. *Sci Rep* 12, 16058, doi:10.1038/s41598-022-20265-w (2022).
